# Age-related characteristics of sedation in pediatric patients and their correlated adverse events: a cohort study

**DOI:** 10.3389/fped.2024.1475891

**Published:** 2024-12-16

**Authors:** Xiaoling Nong, Yixing Lu, Wenqing Jiang, Yanlv Qin, Shunzhong Jing, Tao Chi, Wei Peng, Siyan Liu, Yunan Lin

**Affiliations:** ^1^ Department of Anesthesiology, The First Affiliated Hospital of Guangxi Medical University, Nanning, China; ^2^ Department of Anesthesiology, Maternal and Child Health Hospital of Guangxi Zhuang Autonomous Region, Nanning, China; ^3^ Department of Anesthesiology, Reproductive Hospital of Guangxi Zhuang Autonomous Region, Nanning, China

**Keywords:** children, sedation, age, adverse events, delayed awakening, hypoglycemia

## Abstract

**Objective:**

The objective of this research was to examine the features and potential hazards of sedation in children of varying ages. Additionally, the study aimed to comprehend these variations to enhance the safety and efficacy of clinical applications.

**Methods:**

A retrospective analysis was conducted on case data involving pediatric patients who underwent imaging procedures in outpatient settings and necessitated procedural sedation from 2022 to 2024. The research participants were categorized into three age groups: ≤1 year, 1–3 years, and 3–12 years. The primary sedative agents administered were oral midazolam in conjunction with intranasal dexmedetomidine. We examined the effects of sedation and the occurrence of adverse events across various age groups. Additionally, we applied multivariate logistic regression to identify factors linked to these adverse events.

**Results:**

The study observed 2,194 children, with 879 (40.1%) being ≤1-year-old. The ≤1-year-old group achieved faster sleep onset at 18.7 ± 0.3 min, with no significant variance in awakening time and length of stay among the groups. The incidence of adverse events varied significantly by age, with the highest rate of 12.2% in the ≤1-year-old group and the lowest rate of 9.7% in the 3–12-year-old group. Multivariate analysis revealed age as an independent factor affecting adverse event occurrence, with a relative risk ratio (AOR) of 2.21 (95% CI: 1.31–3.75) for delayed awakening in children ≤1-year-old, 15.03 (95% CI: 1.92–117.61) for hypoglycemia, and a relative risk ratio (AOR) of 4.58 (95% CI: 2.22–9.42) for receiving a significant intervention.

**Conclusions:**

Significant variations in sedation reactions and adverse events were observed across the different age groups. Specifically, children aged ≤1 year exhibited a higher susceptibility to adverse events such as delayed awakening and hypoglycemia.

**Clinical Trial Registration:**

chictr.org.cn identifier (ChiCTR2400082774).

## 1 Introduction

Pediatric sedation is essential to facilitate various medical procedures and ensure patient comfort. As the demand for pediatric sedation increases, younger children are undergoing sedation; however, there is limited awareness of the risks involved in infants and young children. Owing to variations in metabolism and physiological responses, the most effective sedation approaches may differ based on the patient's age. Understanding age-specific attributes and potential risks of sedation in children is essential for enhancing the safety and effectiveness of clinical procedures.

The distinct characteristics of various sedatives has been studied in children. Among these, Ketamine, propofol, midazolam, and dexmedetomidine are the most commonly used. The combination of ketamine and propofol for sedation in children has been shown to have the highest incidence of serious adverse events (1). In contrast, intranasal dexmedetomidine provides better sedation than midazolam with no significant adverse effects (2). Green SM's research indicates that children aged <2 or >13 years are at a higher risk of respiratory adverse events when sedated with ketamine in the emergency department (3). Research also suggests that the likelihood of a reduction in blood oxygen saturation (SPO_2_) during ketamine sedation is comparable between children aged 1–2 years and those older than 2 years, with a reduced incidence of vomiting events observed in the former group (4).

Several studies have explored the sedation issues in children. However, these studies are often restricted to particular age groups or individual drugs, and lack a thorough and systematic evaluation (5–8). Moreover, many of them have limited sample sizes, especially in children under 1-year-old, which restricts the applicability of their findings. Furthermore, the definitions of adverse events and recording criteria varied among the studies, resulting in poor comparability of outcomes. This study systematically evaluated the impact of sedation and its associated negative effects on children of varying age groups through retrospective analysis of data from a sizeable sample. In contrast to prior research, this study distinguishes itself through its extensive sample size and evenly distributed data across different age brackets, thus enhancing the breadth and representativeness of the findings. The primary objective was to examine the effects of sedation and the occurrence of adverse events in various children age groups and to identify factors related to these adverse events.

## 2 Methods

We performed a retrospective cohort analysis of all children who received sedation between March 2022 and March 2024 at the Maternal and Child Health Hospital of Guangxi Zhuang Autonomous Region. This study was registered in the Chinese Clinical Trial Registry (ChiCTR2400082774) and was reviewed and approved by the Medical Ethics Committee of our hospital [No. (2024-3)166]. The parents of the enrolled children signed the informed consent.

### 2.1 Study setting and population

We included children aged 12 years and younger with complete clinical records, documented sedation protocols, and adverse events. Children with prior allergies to sedatives or contrast media, incomplete records, or missing data were excluded. We classified the children into three age groups: ≤1 year, 1–3 years, and 3–12 years.

### 2.2 Outcome measures

The demographic parameters included sex, age, weight, fasting duration, and treatment items. Clinical data included the diagnosis, soothing agents and dosages, and sedation duration. Adverse events consist of (1) Agitation, characterized by abnormal behavioral expressions due to emotional instability. (2) Respiratory adverse events encompass conditions such as apnea (notable spontaneous apnea), upper airway obstruction (characterized by inspiratory retraction or wheezing), and abnormalities in oxygenation (with SPO2 levels dropping to 90% at any given time). (3) An arrhythmia is characterized by an irregular heart rate and/or rhythm. This encompasses bradycardia, which is defined as a heart rate that falls below the normal thresholds for specific age groups: less than 100 beats per minute in infants, under 80 beats per minute in young children, below 70 beats per minute in school-aged children, and fewer than 60 beats per minute in adolescents. (4) Vomiting, specified as the expulsion of gastric contents via the nose or mouth during induction, maintenance of sedation, or emergency department recovery. (5) Delayed awakening, marked by a response time exceeding 90 min to painful stimuli post-sedation. (6) Hypoglycemia was indicated by a random blood glucose level <50 mg/dl. Blood glucose levels were measured during post-treatment monitoring in the resuscitation unit in children who did not wake up or who had been prolonged fasting. The analysis also accounted for cases requiring significant interventions following adverse events, including assisted ventilation, oxygen supplementation, antiarrhythmic or antagonist treatments, and rehydration for hypoglycemia correction. All the data were subjected to double entry by trained researchers to ensure precision and uniformity.

### 2.3 Data quality assurance

All information was sourced from the hospital's outpatient electronic medical records (EMR) to verify its authenticity and reliability. Two researchers independently extracted data from the EMR system and conducted a cross-verification. Regular consistency checks were performed to ensure accuracy and completeness of the entered information. Following data entry, validation was performed through logical and range assessments to filter out incomplete or anomalous data, thereby guaranteeing the reliability of the analyzed dataset.

### 2.4 Statistical analysis

Demographic characteristics, sedation regimen, and the incidence of adverse events were analyzed descriptively for each group. Results are presented as mean ± standard deviation (*x¯* ± *s*) or frequency (percentage) *n* (%). The chi-square (*χ*^2^) test was used to compare the incidence of adverse events during sedation in different age groups of children. The rank sum test (RST) was applied for continuous variables that did not meet the assumption of normal distribution. In addition, multivariate logistic regression was used to examine risk factors for adverse events. Adverse events were considered the dependent variable, whereas age, sex, sedative drug dose, and fasting duration were treated as covariates to assess their impact. The results are reported as ratio of ratios (AOR), 95% confidence intervals (CI), and *p*-values. Statistical analysis was conducted using IBM SPSS Statistics 23.0, where a significance level of *p* < 0.05 was established.

## 3 Results

### 3.1 Baseline characteristics and outcomes

A total of 2,548 children underwent outpatient sedation between March 2022 and March 2024. Of these children, four developed an allergy to the injected contrast during the examination. In addition, 12 children were over 12 years of age. Furthermore, 338 patients with incomplete medical history and follow-up data were excluded from the study. Therefore, the final study population comprised 2,194 pediatric patients, with 879 (40.1%) being children aged 1 year or younger. Male patients represent a greater percentage of all pediatric cases requiring procedural sedation for the purpose of completing examinations, the mean fasting time before sedation in our hospital is 1–2 h. Magnetic Resonance Imaging (MRI) was the most common reason for sedation in children, accounting for 71.8% of cases in children aged 1 year or younger, 88.8% in children aged 1–3 years, and as high as 91.3% in children aged 3–12 years. Auditory Brainstem Response (ABR) testing was also a frequent reason for sedation in children aged 1 year or younger, representing 26.2% of cases. Table 1 provides an overview of the demographic characteristics and medical procedures.

**Table 1:** Characteristics of children by age group.

	≤1 year	1–3 year	3–12 year
	*n* = 879	*n* = 686	*n* = 621
Male *n* (%)	546 (62.1)	484 (70.6)	442 (71.2)
Age (years)	0.46 ± 0.30	2.10 ± 0.66	5.00 ± 1.74
Weight (kg)	7.1 ± 0.1	12.8 ± 0.1	18.4 ± 0.2
Fasting time (h)	0.96 ± 0.03	1.22 ± 0.04	1.75 ± 0.05
Medical procedures *n* (%)			
MRI	631 (71.8)	609 (88.8)	567 (91.3)
CT	11 (1.3)	13 (1.9)	6 (1.0)
ABR testing	230 (26.2)	45 (6.6)	33 (5.3)
Excision of superficial masses	4 (0.5)	17 (2.5)	12 (1.9)
Other	3 (0.3)	2(0.3)	3(0.5)

### 3.2 Evaluation of medication and effects

**Figure 1:**
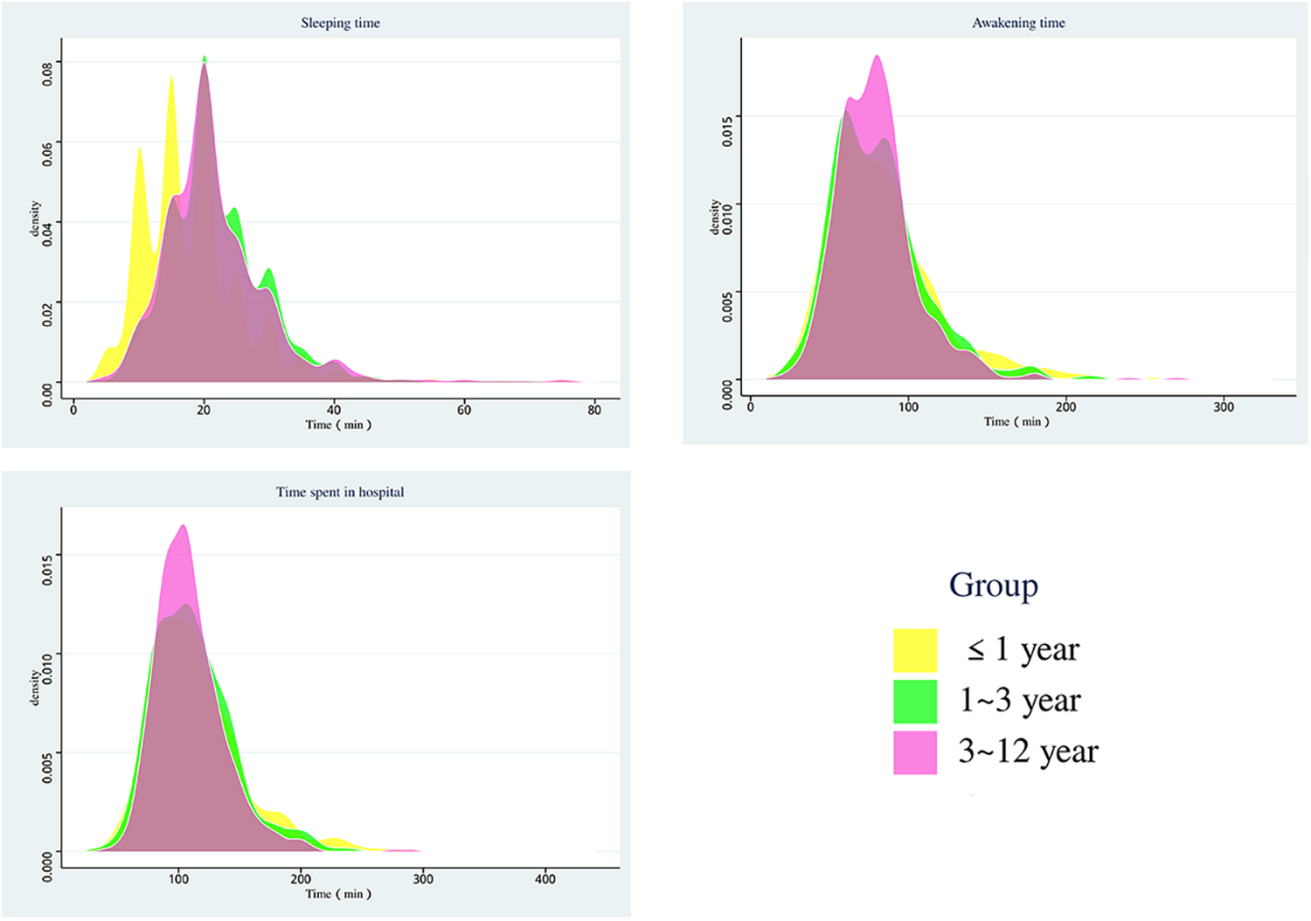
Distribution of sedation time in three groups.

The dexmedetomidine dosage varied significantly across the different age groups (*p* < 0.001). Children in the ≤1 year age group received a mean dose of 2.56 ± 0.03 mcg/kg, while those in the 1–3 and 3–12 year age groups received 2.98 ± 0.03 mcg/kg and 2.96 ± 0.03 mcg/kg, respectively, which were notably higher than the dosage for the ≤1 year age group. There were no significant differences in midazolam dose among the three age groups (*p* = 0.907). The average dose varied 0.46 between 0.47 mg/kg. In terms of time taken to fall asleep, children in the ≤1 year age group took an average of 18.7 ± 0.3 min, which was notably shorter than the 1–3 year age group and the 3–12 year age group (22.0 ± 0.3 min and 21.8 ± 0.3 min, *p* < 0.001). However, there were no significant differences among the three age groups in terms of time to awakening, total length of hospital stay, and success rate of sedation (*p*-values = 0.628, 0.174, and 0.482, respectively) (Figure 1; Table 2).

**Table 2:** Sedation medication and effect evaluation.

	≤1 year	1–3 year	3–12 year	*p*-values
	*n* = 879	*n* = 686	*n* = 621	
Dexmedetomidine (mcg/kg)^a^	2.56 ± 0.03	2.98 ± 0.03	2.96 ± 0.03	<0.001***
Midazolam (mg/kg)	0.47 ± 0.01	0.46 ± 0.00	0.46 ± 0.01	0.907
Sleeping time^b^ (min)^a^	18.7 ± 0.3	22.0 ± 0.3	21.8 ± 0.3	<0.001***
Awakening time^c^ (min)	83.7 ± 1.2	80.7 ± 1.1	79.3 ± 1.0	0.628
Time spent in hospital (min)	115.6 ± 1.4	111.7 ± 1.2	109.1 ± 1.1	0.174
Sedation success rates *n* (%)	827 (94.1)	638 (93.0)	575 (92.6)	0.482

^a^ Significance level of differences between the three groups: ***p < 0.001.

^b^ Time to fall asleep denotes the point at which a patient transitions into sleep following the administration of combination therapy.

^c^ The awakening time denotes the moment when a sedated child exhibits physical movements, opens their eyes, cries, or displays other responses following artificial stimulation. The intervention techniques may involve manipulating the earlobes, pinching the nostrils.

### 3.3 Adverse events

Significant differences existed in the likelihood of requiring critical interventions for post-adverse events across the various age cohorts (*p* < 0.001). The rate of critical interventions was 3.4% for children aged ≤ 1 year, significantly exceeding that of the 1–3 year group (0.1%) and the 3–12 year group (2.3%). No significant difference emerged in the incidence of agitation among the three age groups (*p* = 0.313), with rates fluctuating between 5.7% and 7.4%. Additionally, the occurrence of respiratory depression, arrhythmia, and vomiting showed no significant differences among the three age groups, as evidenced by *p*-values of 0.103, 0.189, and 0.658, respectively. The occurrence of respiratory depression was minimal, with rates between 0.1% and 0.9%. Cardiac arrhythmia appeared rarely, affecting only a small fraction of the ≤1-year-old and 3–12-year-old cohorts. Similarly, vomiting was infrequent, noted solely in the ≤1-year-old and 1–3-year-old groups. Delayed awakening was observed at a rate of 4.0% in children ≤1-year-old, significantly surpassing the rates for the 1–3-year-old (2.8%) and the 3–12-year-old (0.8%) groups. Furthermore, children ≤1-year-old also demonstrated the highest likelihood of hypoglycemia at 1.1%, whereas the occurrence of hypoglycemia was either lower or absent in the 1–3-year-old and 3–12-year-old groups. Statistically significant differences in the incidence of delayed awakening and hypoglycemia among the three age groups were noted, with *p*-values of 0.001 and 0.002, respectively (Table 3).

**Table 3:** Sedation-related adverse events *n* (%).

	≤1 year	1–3 year	3–12 year	*p*-values
	*n* = 879	*n* = 686	*n* = 621	
Agitation	50 (5.7)	50 (7.3)	46 (7.4)	0.313
Respiratory depression	9 (1.0)	1 (0.1)	5 (0.8)	0.103
Arrhythmia	2 (0.2)	0	3 (0.5)	0.189
Vomiting	1 (0.1)	1 (0.1)	0	0.658
Delayed awakening^a^	35 (4.0)	19 (2.8)	5 (0.8)	0.001**
Hypoglycemia^a^	10 (1.1)	0	1 (0.2)	0.002**
Critical interventions^a^	30 (3.4)	1 (0.1)	14 (2.3)	<0.001***
Airway management	9 (1.0)	1 (0.1)	5 (0.8)	
Atropine	1 (0.1)	0	3 (0.5)	
Flumazenil	13 (1.5)	0	5 (0.8)	
Glucose	7 (0.8)	0	1(0.2)	

^a^ Significance level of differences between the three groups: **p < 0.01; ***p < 0.001.

### 3.4 Correlation between age and adverse events

Table 4 presents the findings of the multivariate logistic regression analysis adjusted for age demographics. The analysis revealed that the incidence of respiratory depression in children aged ≤1 year and 1–12 years was 1.0% and 0.5% (AOR 2.24, 95% CI: 0.80–6.32, *p* = 0.127), respectively. For cardiac arrhythmia, the incidence was 0.2% for both age groups (AOR 0.50, 95% CI: 0.05–4.77, *p* = 0.543). Vomiting occurred at 0.1% in each group (AOR 1.49, 95% CI: 0.09–23.81, *p* = 0.779). After controlling for additional factors, age did not significantly influence the incidences of respiratory depression, arrhythmia, or vomiting. Conversely, the incidence of delayed awakening was 4.0% and 1.8% (AOR 2.21, 95% CI: 1.31–3.75, *p* = 0.003). Hypoglycemia was observed in 1.1% and 0.1% (AOR 15.03, 95% CI: 1.92–117.61, *p* = 0.010). The likelihood of receiving a significant intervention was 3.4% and 1.1% (AOR 3.04, 95% CI: 1.63–5.69, *p* < 0.001). Age significantly affected the incidence of delayed awakening, hypoglycemia, and significant interventions when controlling for other factors.

**Table 4:** Relationship between age and sedation adverse events.

	≤1 year	1–12 year	AOR (95% CI)	*p*-values
	*n* = 879	*n* = 1,307		
Agitation	50 (5.7)	96 (7.3)	0.76 (0.54, 1.08)	0.129
Respiratory depression	9 (1.0)	6 (0.5)	2.24 (0.80, 6.32)	0.127
Arrhythmia	2 (0.2)	3 (0.2)	0.50 (0.05, 4.77)	0.543
Vomiting	1 (0.1)	1 (0.1)	1.49 (0.09, 23.81)	0.779
Delayed awakening^a^	35 (4.0)	24 (1.8)	2.21 (1.31, 3.75)	0.003**
Hypoglycemia^a^	10 (1.1)	1 (0.1)	15.03 (1.92, 117.61)	0.010*
Critical interventions^a^	30 (3.4)	15 (1.1)	3.04 (1.63, 5.69)	<0.001***

^a^ Level of significance of differences between ≤1 -year-old and 1–12 -year-old groups: *p < 0.05; **p < 0.01; ***p < 0.001.

## 4 Discussion

In this study of 2,194 patients, children aged one year or younger comprised 40.1%. The data indicated that male children exhibited a higher likelihood of requiring sedation, potentially due to the more pronounced clinical symptoms often observed in boys. Additionally, children undergoing elective sedation did not adhere to strict fasting protocols; most fasted for 1–2 h. Children aged one year or younger had the shortest fasting duration, averaging 0.96 h, while those aged 3–12 years fasted the longest, with an average of 1.75 h. Some studies suggest that no correlation exists between fasting duration and adverse events related to sedation (9–11). Is routine fasting essential for sedation procedures conducted outside of the operating room? The question of whether a standard prohibition on these practices remains an open debate (12). The correlation between fasting duration and negative outcomes, including vomiting and aspiration during sedation, requires further prospective clinical investigations. For the sake of safety and comfort, we advise that pediatric patients undergoing sedation in an outpatient setting should refrain from eating for a minimum of 2 h prior to the procedure. MRI examinations frequently necessitate sedation in children, particularly older cohorts, where the examination rate reaches 91.3% among those aged 3–12 years. Furthermore, for children aged one year or younger, Auditory Brainstem Response (ABR) testing also commonly requires sedation, with an occurrence rate of 26.2%.

Dexmedetomidine is increasingly replacing conventional sedative agents such as chloral hydrate and sodium phenobarbital, demonstrating enhanced safety and reliability compared to its counterparts (13, 14). Several research investigations have shown the practicality and safety of administering midazolam in conjunction with dexmedetomidine for pediatric sedation in diagnostic and therapeutic procedures (15, 16). In alignment with our clinical experience, midazolam and dexmedetomidine are the primary sedative agents utilized in this study. Dexmedetomidine achieves sedative and analgesic effects via α_2_-adrenergic receptor activation, whereas midazolam, a benzodiazepine, enhances GABAergic activity to induce sedation. Although sedation success rates for both agents were similar across diverse age groups, the findings indicated greater sensitivity to dexmedetomidine in infants and young children, with effective sedation attainable at lower doses (*p* < 0.001). In contrast, midazolam dosage showed no significant variance among the age groups. Thus, precise adjustment of the dexmedetomidine dosage in younger children is essential to ensure both safety and efficacy.

The study's findings indicate that children aged one year or younger experienced shorter sleep onset times, averaging 18.7 min. This duration was significantly lower (*p* < 0.001) than the sleep onset times in the 1–3-year-old (22.0 min) and 3–12-year-old group (21.8 min) groups. The faster response to sedative medications in younger children may stem from their unique metabolic and physiological traits. Despite shorter sleep duration, these children had relatively longer awakening periods, averaging 83.7 min. In comparison, the awakening times for the 1–3-year-old and 3–12-year-old groups were 80.7 min and 79.3 min, respectively, with no significant differences observed among the three groups (*p* = 0.628). Although younger children demonstrated faster sleep onset, the effects of sedatives might persist longer, necessitating careful monitoring during the awakening phase in clinical settings. Differences in sleep onset and awakening times existed among the various age groups; however, the overall length of stay remained largely uniform. Effective sedation was achieved across all ages, with no statistically significant variation in the sedation success rate among groups (*p* = 0.482), although children aged one year or younger exhibited the highest success rate at 94.1%).

Although sedative medications are generally safe and effective, some children still experience adverse events. Agitation is one of the most prevalent complications associated with sedation in pediatric patients (17) and emerged as the most common adverse event in this investigation. However, the occurrence of abnormal agitation remained consistent across the various age groups (*p* = 0.313). Previous research has highlighted respiratory events and vomiting as frequent adverse effects of ketamine sedation in children (1, 3). In contrast, the present study recorded a low incidence of respiratory depression (15 cases), arrhythmia (5 cases), and vomiting (2 cases) when dexmedetomidine was used in conjunction with midazolam. Active symptomatic management has contributed to improving these children's conditions and mitigating serious consequences. Variations in sedative drug outcomes stem from differences in physicochemical properties, resulting in a notable disparity in the incidence of adverse effects by drug type (18). Research indicates that the intranasal administration of dexmedetomidine in infants and young children can extend recovery time following sedation. In contrast, children receiving inhaled sevoflurane alone tend to fall asleep and recover more rapidly; however, the brief duration of sedation maintenance limits its application to short-term procedures such as CT scans and echocardiography. Given that this investigation is retrospective and features relatively consistent medication protocols, there is a need for prospective studies to elucidate the variances in sedative efficacy and associated risks among patients administered different sedatives. When stratified by age, our findings reveal that children under one year old necessitate a higher incidence of critical interventions (3.4%), which is significantly greater than that observed in other age cohorts (*p* < 0.001). These interventions encompass oxygen therapy, fluid resuscitation, and the administration of reversal agents. Notably, hypoglycemia and delayed awakening occurred more frequently in the ≤1 year age group, with hypoglycemia affecting 1.1% of these children, while the other age cohorts exhibited virtually no occurrences (*p* = 0.002).

Multivariate logistic regression analysis indicated that children aged one year or younger had a significantly elevated risk of hypoglycemia compared to other age groups (AOR 15.03, 95% CI: 1.92–117.61, *p* = 0.010). In infants who are unable to communicate effectively, blood glucose levels that fall below the normal neurogenic response threshold [<60 mg/dl (3.3 mmol/L)] necessitate a thorough evaluation and intervention. In older children, a blood glucose measurement of <55 mg/dl (<3.0 mmol/L) prompts a search for food or assistance, while cognitive function is compromised when levels drop below <50 mg/dl (<2.8 mmol/L). For caution, the minimum blood glucose threshold applicable to all infants and children is set at 50 mg/dl (19, 20). Energy metabolism alterations during fasting in normal 2–3 day postnatal neonates, infants, and children did not display significant differences from adult patterns. However, owing to the relatively larger brain volume in infants and young children compared to their body weight, the brain's energy demands are considerably higher. Glucose utilization per kilogram of body weight is 2–3 times greater in this population than in adults (4–6 mg/kg/min) (21). Consequently, plasma glucose concentrations decline more rapidly, leading to rapid onset of hyperketonemia. Therefore, when infants and young children undergo prolonged fasting during sedation examinations, glycogen stores diminish quickly, resulting in acute metabolic disturbances and, consequently, hypoglycemia. While short-term hypoglycemia typically resolves completely, severe and prolonged episodes may result in lasting brain damage (20, 22). The hypoglycemic incidents documented in this study primarily affected neonates and preterm infants at high risk who did not actively seek food or assistance to combat hypoglycemia, despite demonstrating a neurogenic response, including apathy, coma, and slow respiratory rhythm. Hence, vigilance is crucial for both infants and young children. It is essential to limit pre-sedation fasting durations, monitor blood glucose levels in cases of extended awakening times, and provide rehydration therapy when needed. Additionally, applying Whipple's triad (23) can help to confirm the presence of hypoglycemia in these children for enhanced management and treatment. The criteria include: (1) the existence of distinct clinical symptoms, (2) occurring alongside accurately measured low plasma glucose levels using sensitive and precise techniques, and (3) the resolution of clinical signs within minutes to hours after normoglycemia is restored (24).

The study revealed a notably increased rate of delayed awakening in children aged one year or less (AOR 1.86, 95% CI 1.19–2.92, *p* = 0.007). The sedative agents examined were mainly processed in the liver and their metabolites were eliminated through the kidneys. It is important to note that most metabolic enzymes are not fully developed at birth, but mature throughout childhood (25). Hepatic clearance relies heavily on intrinsic clearance, which is affected by developmental variations in the expression and activity of enzymes. One key enzyme in drug metabolism is CYP3A4, yet neonates exhibit less than 10% of adult CYP3A4 activity. Other drug-metabolizing cytochrome CYP isoenzymes, such as CYP2E1, CYP2D6, CYP2C9, and CYP2C19, also show reduced maturity in neonates compared to adults (26). Typically, renal development concludes by age 2, and the glomerular filtration rate (GFR) in neonates is only 30%–40% of adult levels. This rate increases swiftly after birth, achieving 50%–60% of the adult GFR by roughly the third week. Following this period, steady increases occurred, reaching adult levels within the first year. Despite differences in body size, GFR values in children remain lower than in adults (27). Consequently, drug metabolism, clearance, and excretion are slower in infants and young children, resulting in extended drug effects and longer awakening times.

This study highlights the significance of age-specific considerations when developing sedation protocols for pediatric patients. Infants (≤1 year) display distinct pharmacokinetic characteristics and are more vulnerable to adverse effects such as delayed recovery and hypoglycemia. For infants, it is crucial to minimize fasting times, enhance monitoring after sedation, and provide timely glucose supplementation to prevent hypoglycemia and associated neurological issues. Additionally, selection of short-acting and rapidly metabolizing sedative agents can help mitigate the risk of delayed recovery.

This study constituted a single-center retrospective analysis utilized data from the hospital's electronic medical record (EMR) system. Given this singular data source, concerns regarding selection bias arose, potentially limiting the generalizability of the findings. The research depended on retrospectively gathered electronic case records, and variations in detail and accuracy of EMR entries may have occurred, influenced by physicians' documentation practices and accessibility of patient information. Despite the inclusion of multiple potential confounders (age, sex, and fasting duration) in the logistic regression analysis, unmeasured or inadequately adjusted confounding variables may persist due to data constraints. It is important to note that this retrospective observational design emphasizes associations rather than causality. Although a significant relationship was observed between age and both sedation success and adverse events, the impact of other potential factors remains uncertain. Future prospective studies, such as randomized controlled trials (RCTs), could enhance the validation of these findings and provide a clearer understanding of causal relationships.

## 5 Conclusion

In summary, this study comprehensively examined the properties of sedation and its associated adverse effects in children of varying ages. This highlights the considerable impact of age on the efficacy and safety of sedation, offering findings that are both indicative and applicable. It is advisable for clinical practitioners to consider age when administering sedation in children and to modify sedation protocols based on the outcomes of this study to enhance safety and effectiveness.

## Data Availability

The datasets presented in this study can be found in online repositories. The names of the repository/repositories and accession number(s) can be found below: DOI: 10.6084/m9.figshare.26384485.
